# Image Quality Assessment and Reliability Analysis of Artificial Intelligence-Based Tumor Classification of Stimulated Raman Histology of Tumor Biobank Samples

**DOI:** 10.3390/diagnostics14232701

**Published:** 2024-11-30

**Authors:** Anna-Katharina Meißner, Tobias Blau, David Reinecke, Gina Fürtjes, Lili Leyer, Nina Müller, Niklas von Spreckelsen, Thomas Stehle, Abdulkader Al Shugri, Reinhard Büttner, Roland Goldbrunner, Marco Timmer, Volker Neuschmelting

**Affiliations:** 1 Department of General Neurosurgery, Center for Neurosurgery, Faculty of Medicine and University Hospital Cologne, University of Cologne, 50937 Cologne, Germany; 2 Institute for Neuropathology, University of Duisburg-Essen, 45141 Essen, Germany; 3 Department of Neurosurgery, Westküstenklinikum Heide, 25746 Heide, Germany; 4 Institute for Neuropathology, Faculty of Medicine, University Hospital Cologne, University of Cologne, 50937 Cologne, Germany; 5 Department of Pathology, Faculty of Medicine, University Hospital Cologne, University of Cologne, 50937 Cologne, Germany

**Keywords:** stimulated Raman histology, artificial intelligence, digital pathology, brain tumors

## Abstract

Background: Stimulated Raman histology (SRH) is a label-free optical imaging method for rapid intraoperative analysis of fresh tissue samples. Analysis of SRH images using Convolutional Neural Networks (CNN) has shown promising results for predicting the main histopathological classes of neurooncological tumors. Due to the relatively low number of rare tumor representations in CNN training datasets, a valid prediction of rarer entities remains limited. To develop new reliable analysis tools, larger datasets and greater tumor variety are crucial. One way to accomplish this is through research biobanks storing frozen tumor tissue samples. However, there is currently no data available regarding the pertinency of previously frozen tissue samples for SRH analysis. The aim of this study was to assess image quality and perform a comparative reliability analysis of artificial intelligence-based tumor classification using SRH in fresh and frozen tissue samples. Methods: In a monocentric prospective study, tissue samples from 25 patients undergoing brain tumor resection were obtained. SRH was acquired in fresh and defrosted samples of the same specimen after varying storage durations at −80 °C. Image quality was rated by an experienced neuropathologist, and prediction of histopathological diagnosis was performed using two established CNNs. Results: The image quality of SRH in fresh and defrosted tissue samples was high, with a mean image quality score of 1.96 (range 1–5) for both groups. CNN analysis showed high internal consistency for histo-(Cα 0.95) and molecular (Cα 0.83) pathological tumor classification. The results were confirmed using a dataset with samples from the local tumor biobank (Cα 0.91 and 0.53). Conclusions: Our results showed that SRH appears comparably reliable in fresh and frozen tissue samples, enabling the integration of tumor biobank specimens to potentially improve the diagnostic range and reliability of CNN prediction tools.

## 1. Introduction

Stimulated Raman Histology (SRH) is a new label-free chemical optimal imaging method for near real-time intraoperative analysis of fresh tissue samples using stimulated Raman scattering (SRS) microscopy [1]. A small unprocessed tissue sample is scanned in a portable SRH microscope equipped with a dual-wavelength fiber laser at two Raman shifts, corresponding to the intrinsic biological properties of lipids, DNA, and proteins [1,2]. The SRS image is converted into a virtual image (SRH image) resembling H&E-like staining to facilitate clinical interpretation [1]. The entire process of image acquisition takes 2–3 min, enabling improvements in intraoperative diagnosis, which is integral to tumor surgery, biopsy, and resection control. SRH has been clinically validated against standard H&E sections and has proven to be a valuable time-saving intraoperative tool for diagnosing the main adult and pediatric neurooncological tumor types [3,4,5,6,7,8,9]. Furthermore, SRH imaging has been shown to be feasible for detecting residual tumor tissue in the infiltration zone of diffuse gliomas [10]. One limitation of these studies is that automated, observer-independent real-time image analysis was not feasible for all neurooncological tumor entities with adequate predictive performance. The necessity for visual assessment of SRH images, which is time-consuming, continues to limit its use in routine surgical settings. 

Recent studies addressing this issue have shown that the analysis of SRH images with Artificial Intelligence (AI) methods reveals promising results for fully automated, time-efficient intraoperative diagnosis [11,12]. A Convolutional Neural Network (CNN) trained with SRH images from different neurooncological tumors achieved valid results for distinguishing tumor from normal brain tissue and can be used for intraoperative resection control [13]. Furthermore, recent studies by Hollon T et al. demonstrated promising results for predicting the histopathological diagnosis of the main classes of neurooncological tumors, with a high overall diagnostic accuracy of 94.6% [14], and for classifying molecular subtypes of diffuse adult gliomas [15]. Moreover, a CNN-based classifier showed good diagnostic performance in distinguishing vital tumor tissue from therapy-associated changes and pseudoprogression [16]. 

While current studies on SRH and AI tools have achieved good results in predicting common tumor classes, the reliable prediction of rarer tumor entities remains limited, most likely due to the lower representation of rare tumor samples in the training and test datasets. Some rare tumor classes have not yet been represented in any SRH-based AI tool. Therefore, the routine clinical use of AI analysis for SRH is not yet feasible. The availability of broader, more diverse, and more general diagnostic tools for diverse classification tasks would be highly beneficial to the clinical workflow in neurooncological surgery. 

To train new AI tools and improve existing ones, large datasets for training and validation are necessary, especially for rare tumor entities and molecular tumor subclasses. SRH is a relatively new technology, and SRH microscopes are not broadly available. Moreover, rare neurooncological tumors are few in number due to the generally low incidence of these diseases. Therefore, even pooling data from different centers with access to SRH microscopes might not provide a sufficient dataset for reliable model training in a reasonable timeframe. Recent studies using only fresh specimens have therefore lacked the opportunity to implement larger training and test datasets. 

One option to overcome this obstacle could be to augment the training data pool with SRH images obtained from frozen tumor tissue stored in research biobanks. The aim of this study was to assess the image quality of SRH analysis in previously frozen tissue samples and to perform a reliability analysis of CNN-based entity and molecular tumor class prediction using SRH in fresh and defrosted samples of the same specimen. The findings were validated in a randomly selected brain tumor biobank cohort with varying freezing durations. Data analyses of previously frozen samples subjected to SRH are scarce. This study assesses the applicability of SRH in defrosted tumor tissue and offers an opportunity to incorporate the use of frozen tissue into the workflow of developing SRH-based diagnostic tools. 

## 2. Materials and Methods

### 2.1. Patient Dataset

In a single-center prospective observational study, patients were recruited in 2022 and 2023 after surgical resection of a brain tumor, indicated by an interdisciplinary neuro-oncological tumor board. The study was conducted according to the guidelines of the declaration of Helsinki and approved by the Ethics Committee of the University Hospital Cologne, Germany, (IRB No. 21-1238). All patients gave their written informed consent for the scientific use of their data.

Inclusion criteria were (1) suspected tumor, (2) age over 18, (3) patient is willing and able to give informed consent for participation. Patients were excluded if not willing or able to give informed consent for participation. Final histopathological results were obtained following routine clinical pathology diagnostics.

### 2.2. Biobank Dataset

Frozen tissue samples of varying storage duration were randomly selected from the local neurooncology research biobank to undergo SRH acquisition and analysis. Of note, small samples of every specimen that had been contributed to the biobank for storage had been scanned in the SRH microscope prior to this study at the time of the initial tissue collection and discarded after imaging as part of the routine research tissue collection process. The initial SRH imaging data of the specimens served as reference for the data acquired in the study. The observers and reviewers acquiring and analyzing the SRH images were blinded for the histopathological diagnosis and storage duration. All patients had given their written informed consent for acquisition, storing and scientific use of the tissue samples (Ethics Committee of the University Hospital Cologne, Germany, IRB No. 03-170). 

### 2.3. Specimen Collection and Stimulated Raman Histology

During surgery, a small tissue sample was collected from the core of the lesion for SRH imaging. The sample #1 (3–4 mm in size) of the specimen was processed immediately and a fresh unstained squash preparation on a microscopic glass slide with a coverslip was prepared. The slide was scanned (scan 1 sample #1) with a field-of view of 2.7 × 2.9 mm in a clinical SRH microscope (Invenio Imaging Inc., Santa Clara, CA, USA). The portable SRH microscope contains four main components: [1] a fiber-coupled microscope, [2] a dual-wavelength (790 and 1020 nm) fiber-laser module, [3] a laser and microscope control module, and [4] a computer for data displaying, processing, and application of a convolutional neuronal network [1,17]. The power in focus is approximately 120 and 160 mW for the 790 and 1020 nm beam, respectively [17]. Tissue samples were scanned at two predefined Raman Shift wave numbers, characteristic for the vibrational frequencies of lipid-rich structures (2845 cm^−1^), proteins and DNA (2930 cm^−1^). The image contrast is generated by the differences in the Raman scattering spectrum depending on the tissue composition. The raw images were stitched and via subtraction a virtual H&E like image was created digitally, referred to as the SRH image as previously described in detail [1,13,17,18,19,20]. 

The squash preparation of sample #1 was then immediately frozen at −80 °C and stored in a regular deep-freezer for research purposes. After varying time intervals (range 2–164 days) the sample was slowly defrosted under room temperature conditions. 

**Figure 1:**
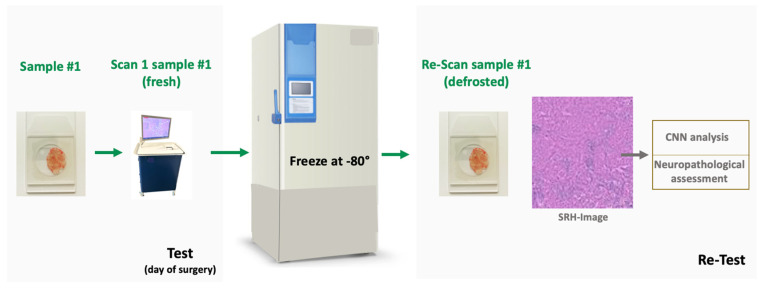
Workflow of the test–retest analysis of the patient dataset. A small (3-4 mm) tissue sample (sample #1) was collected during surgery and immediately processed for SRH imaging. The fresh squash preparation was scanned in the SRH microscope (scan 1 sample #1) and frozen at −80 °C afterwards. After varying time intervals, the sample was defrosted and scanned again in the SRH microscope (re-scan sample #1). All SRH images from fresh and frozen samples were assessed for image quality and occurrence of freezing artifacts by an experienced neuropathologist and analyzed by the CNNs. CNN: Convolutional Neural Network.

After defrosting sample #1 was scanned again (re-scan sample #1) in the SRH microscope with a field-of view of 2.7 × 2.9 mm (see Figure 1). 

The tissue samples from the biobank dataset (5–10 mm in size) were deep frozen with liquid nitrogen without further agents for cryoconservation immediately after resection on the day of surgery and stored in the local tissue biobank at −80 °C. Tissue samples with various storage duration were slowly defrosted at room temperature and scanned in the SRS microscope using a small sample of 2 × 2 mm in size and a field-of view of 2.7 × 2.9 mm. For all biobank specimens a previously acquired SRH image scanned from a different fresh tissue sample of the same specimen on the day of surgery was available for comparison. All SRH images were analyzed by the CNNs.

### 2.4. Image Quality Assessment

All SRH images in the patient dataset were reviewed as DICOM images with 3600 × 3900 pixels in a random order by a senior board-certified neuropathologist with more than 16 years of experience in the field. The pathologist was blinded for the histopathological diagnosis, clinical data and the timepoint of the SRH scan (fresh vs. defrosted). Images were classified according to common criteria applied in digital pathology with scores ranging from 1–5 (1 indicating the best result and 5 the worst result) for the following parameters: (freezing) artifacts, general image quality, image sharpness and color contrast [21,22]. Mean values and standard deviation for each parameter were calculated for fresh and defrosted samples.

### 2.5. Image Analysis by Convolutional Neural Networks

For prediction of the histo- and molecular pathological tumor classes, two established and prospectively validated CNNs were used for inference, as previously described in detail [14,15]. In brief, in the first network, overlapping high-resolution patches from the SRH images were generated by a sliding window algorithm and used for training of the deep CNN. The CNN was trained to classify 13 histological categories: White matter, gray matter, gliosis, ependymoma, malignant glioma, diffuse low grade glioma, medulloblastoma, lymphoma, pilocytic astrocytoma, meningioma, metastasis, pituitary adenoma. For the final diagnostic prediction, all patches from one specimen were merged into a probability distribution map [14]. The second network was trained to classify molecular diagnostic and prognostic features in gliomas: the IDH mutation and the LOH status as previously described [15]. In brief, an SRH image feature encoder and a genetic embedding model using public glioma genomic data were pretrained. Both models were then integrated into a single transformer architecture for multilabel molecular glioma classification [15]. Both networks are integrated in the SRH microscope analysis software and were applied for inference as previously published.

#### Statistical Analysis

Statistical analysis was performed using SPSS Statistics Version 29 (IBM, Chicago, IL, USA). For descriptive statistics, continuous values are given in mean with standard deviation.

Internal consistency of the CNN prediction was analyzed using Cronbach’s alpha (Cα). Cronbach’s alpha is a well-established statistical measure [23] used to evaluate the internal consistency or reliability of a set of scale items. It assesses how closely related a set of items are as a group, indicating the extent to which they measure the same underlying construct.

## 3. Results

### 3.1. Prospective Patient Dataset—Characteristics

We included 25 consecutive patients undergoing brain tumor surgery for different tumor entities. The most common neurooncological tumor classes (diffuse adult gliomas, meningioma, pituitary adenomas, various carcinoma and melanoma metastases, schwannoma) were represented. A total of 25 tumor samples were collected and 50 SRH images were acquired. The mean interval between the day of surgery and the day of thawing was 36 days (range 2–164) (see Supplementary Materials Table S1).

### 3.2. Biobank Dataset—Characteristics

The biobank dataset comprised 30 randomly chosen tissue samples from the tissue biobank, representing the most common tumor classes (diffuse adult glioma, meningioma, pituitary adenoma, schwannoma, metastasis). Different samples from all specimens had been scanned in the SRH microscope during the initial surgery. A total of 60 SRH images were acquired. Mean storage duration was 26 months (±12 months).

### 3.3. Image Quality Score

**Figure 2:**
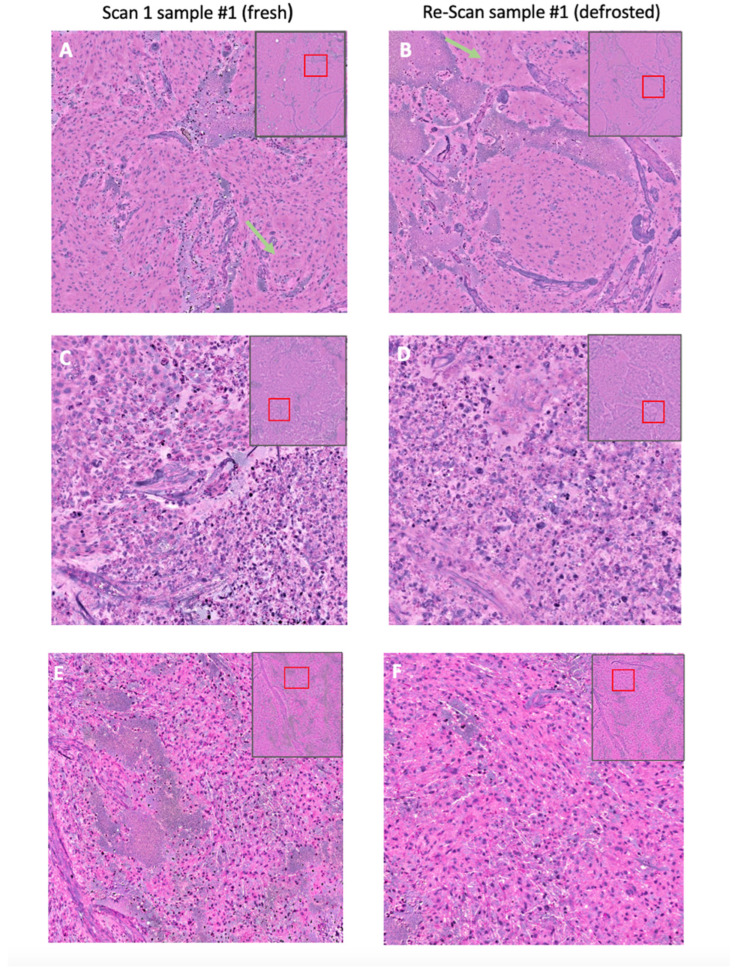
SRH images of fresh and thawed tissue samples. Upper row: SRH images of a meningioma (CNS WHO grade 1) ((**A**): scan 1 sample #1, fresh; (**B**) re-scan 1, sample #1, defrosted), showing typical histologic features, such as meningothelial whorls (green arrows). Middle row: SRH images of a pulmonary adenocarcinoma metastasis ((**C**) scan 1, sample #1, fresh; (**D**) re-scan 1, sample #1, defrosted), showing sheets of epithelial tumor cells. Lower row: SRH images of a glioblastoma, IDH wild type (CNS WHO grade 4) ((**E**) scan 1, sample #1, fresh; (**F**) re-scan 1, sample #1, defrosted), showing infiltration of fibrillary tumor.

All SRH images of fresh and frozen tumor samples from the prospective patient dataset were visually assessed for image quality, image sharpness, color contrast, and occurrence of potential freezing artifacts by the same experienced neuropathologist. All scores ranged from 1–5, with 1 indicating the best and 5 the worst result. Typical histologic features of the tumor classes were identified in the SRH images of both fresh and defrosted samples (see Figure 2).

Mean total image quality score for all SRH images was 1.96 ± 0.95. There was no difference in the mean image quality score between the SRH images of fresh and defrosted samples (2.08 ± 0.99 vs. 1.84 ± 0.99). Mean image sharpness and color contrast showed equivalent values of 2.12 ± 0.85 and 2.26 ± 085, respectively. No significant differences were observed between the values of the fresh and frozen samples. Relevant artifacts (score ≥ 3, range 1–5, 1 indicating no artifacts, 5 severe artifacts), potentially due to freezing, were described in 5 out of 25 (20%) SRH images of defrosted tissue samples. Artifacts in SRH images of freshly processed specimens were observed in 6 out of 25 cases (24%).

We further assessed the occurrence of freezing artifacts depending on the storage duration in an additional subset of 5 biobank specimens (one from each class: meningioma, pituitary adenoma, diffuse glioma, schwannoma, and metastasis), with freezing periods ranging from 86 to 92 months and a mean freezing period of 90 months. No relevant freezing artifacts were observed. Mean image quality score was comparable to the patient dataset with a mean storage duration of 36 days (2.2. vs. 1.96). For these specimens, no SRH images of fresh specimens were available for further CNN classification due to the long storage duration of approximately 8 years, as SRH had not yet been available in clinics at that time.

### 3.4. Prospective Patient Dataset—CNN Based Histological Tumor Class Prediction

Among all scans, after defrosting compared to the corresponding fresh specimen, 24 out of 25 (96%) SRH images were classified correctly within the same diagnostic subgroup for intraoperative distinction and decision-making (tumor, non-tumorous, non-surgical tumors such as lymphoma, surgical tumors with glial and non-glial entities) according to the tumor group classification model by Hollon TC et al. [14]. The CNN predicted the same histological tumor class in 92% of SRH images (23/25) of thawed samples.

**Figure 3:**
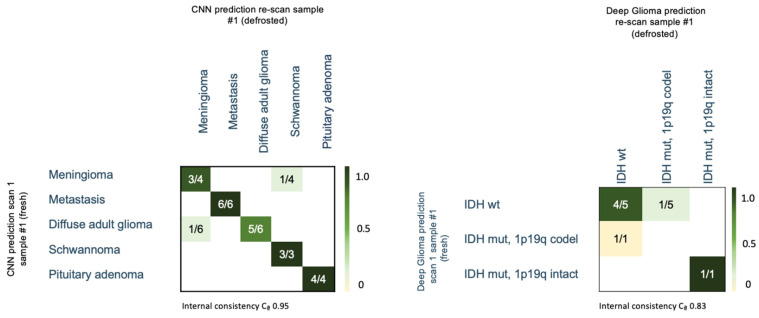
Confusion matrix of the CNN-based histological entity differentiation (**left**), and diffuse adult-type glioma subclassification (**right**) in fresh and frozen tumor tissue samples from the same patient. Ca = Cronbach’s alpha.

A change in CNN diagnosis of SRH images after defrosting occurred in one patient with glioma, which was classified as meningioma, and one patient with meningioma, classified as schwannoma (see Figure 3). No classification change was associated with potential freezing artifacts. The internal consistency (scan sample #1 vs. re-scan sample #1) was high, with a Cronbach’s alpha (Cα) of 0.95.

### 3.5. Prospective Patient Dataset—CNN Based Molecular Subtype Classification (“Deep Glioma”)

SRH images of the six predicted adult-type diffuse glioma specimens underwent CNN-based molecular subtype classification. The CNN predicted the same molecular subtype after defrosting in 67% (4/6) of glioma cases compared to the initial diagnosis on fresh tissue and showed good internal consistency with a Cα of 0.83 (see Figure 3). Discordant classification occurred in one IDH wildtype glioma after defrosting, as well as in one fresh specimen of an IDH wildtype glioma, which was misclassified as oligodendroglioma (IDH mut, 1p19q codel) in scan 1. Prediction levels were comparably low in these borderline cases, ranging from 49–67%.

### 3.6. Biobank Dataset—CNN Based Histological Tumor Class Prediction

**Figure 4:**
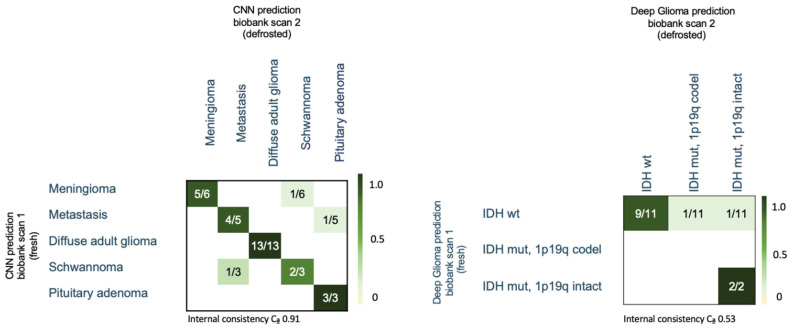
Confusion matrix of the CNN-based histological entity differentiation (**left**), and diffuse adult-type glioma subclassification (**right**) in tumor biobank samples comparing SRH images of fresh and frozen tumor samples. Ca = Cronbach’s alpha.

The CNN predicted the same entity diagnosis in 27 out of 30 (90%) cases in the biobank dataset (see Figure 4). A change in CNN diagnosis of SRH images after defrosting occurred in one patient with schwannoma, which was classified as metastasis; one patient with meningioma, which was classified as schwannoma; and one patient with metastasis, which was classified as pituitary adenoma (see Figure 4). The internal consistency was high, with a Cα of 0.91.

We further assessed the occurrence of classification changes depending on the storage duration. In the biobank dataset, freezing periods ranged from 3 to 38 months, with a mean storage duration of 26 months. Moreover, 17 specimens had a freezing period of over two years and 13 specimens had a storage duration less than 2 years. We did not observe significant differences in the tumor entity classification for longer freezing periods. In the group of specimens with a freezing period of over two years, two changes in tumor entity classification were observed (11.8%), whereas for the group with freezing duration less than two years, likewise two classification changes (15%) were detected.

### 3.7. Biobank Dataset—CNN-Based Molecular Subtype Classification (“Deep Glioma”)

SRH images of the 13 predicted adult-type diffuse glioma specimens in the biobank dataset underwent CNN-based molecular subtype classification. The CNN predicted the same molecular subtype after defrosting in 85% (11/13) of glioma cases compared to the initial diagnosis on fresh tissue (see Figure 4). Discrepant classification occurred in two IDH wildtype gliomas classified as IDH mutant. Prediction levels were comparably low in these borderline cases. Therefore, internal consistency was lower, with a Cα of 0.53.

## 4. Discussion

Reliable intraoperative histolopathological diagnosis is essential to achieve maximum safe tumor resection, especially for lesions in eloquent areas, cases with previously unknown diagnosis, or inconclusive intraoperative findings [24]. SRH, in combination with CNN-based analyses, has shown promise as a time-efficient tool for intraoperative diagnosis of the main classes of neurooncological tumors [3,4,13,14,25]. Established CNNs are trained on large datasets containing common tumor entities, but rare cases—which might lead to inconclusive intraoperative findings and difficult surgical decisions—are underrepresented due to their low incidence. One potential solution to address this gap is to augment available datasets with frozen tissue samples from tissue biobanks. Raman spectroscopy has previously been successfully performed on formalin-fixed and paraffin-embedded (FFPE) tissue [26], as well as on frozen sections [27,28] of glioblastoma, to distinguish vital tumor, necrosis, and peritumoral regions with classification accuracy rates ranging from 70% to 99% [26,27,28]. These findings indicate that histological features of neurooncological tumors can be captured adequately in spectroscopic images even after freezing. However, extensive tissue processing—such as formalin fixation and paraffin embedding—can cause severe changes in spectral properties [26,29,30], leading to potential misclassification. In contrast, the freezing process does not interfere with chemical properties, suggesting that spectroscopic images of defrosted tissue samples remain stable. However, the applicability of SRH and CNN analyses on frozen and defrosted tissue, as well as on FFPE samples, was unclear and may substantially influence the accuracy of the CNN models.

The first finding of our study is that SRH is feasible in defrosted tumor samples, with good image quality and no significant freezing artifacts, as confirmed by visual neuropathological analysis. High general image quality, good color contrast, and sharpness—common quality parameters in digital pathology—are essential for visual analysis of SRH images of defrosted tissue samples and are prerequisites for subsequent CNN analyses.

In conventional intraoperative histomorphological frozen section analysis, artifacts are often reported due to several steps during section preparation, including mechanical tissue destruction, tissue preprocessing, insufficient freezing stability, mechanical sectioning, and slide preparation [27,31,32]. The occurrence of artifacts may complicate a timely and reliable diagnosis, delaying surgical procedures. In comparison, SRH, an optical method based on infrared spectroscopy, can be performed on samples with a thickness of several millimeters, which are less susceptible to tears compared to conventional microscopy of thin frozen sections. Furthermore, SRH requires fewer tissue processing steps, reducing the risk of artifact occurrence, as confirmed in our study.

Tissue storage at −80 °C is a commonly used technique for conservation of biospecimens with known high-quality preservation of proteins and DNA over years to decades [33,34]. As SRH mainly relies on the intrinsic properties of lipids, DNA, and proteins, tissue storage at −80 °C appears feasible, even for long periods, as observed in our study samples stored for over two years. Further analysis of a subset of five biobank specimens with freezing periods of up to eight years showed no relevant freezing artifacts and image quality comparable to the patient dataset with a mean freezing period of 36 days. This suggests that SRH imaging is feasible with high quality, regardless of storage duration.

This finding aligns with a comparative study in conventional histopathology on brain tissue specimens stored at −80 °C, which investigated the effect of long-term frozen storage on immunohistochemistry analyses. Their multiple regression analysis revealed general stability of the examined antigens over decades [35]. Considering this finding, analysis of molecular tumor subtype using SRH in biobank specimens seems to be technically feasible.

The second aim of our study was to evaluate the reliability of an established CNN in both fresh and previously frozen tumor samples to enable potential data augmentation for future AI training. Our results demonstrate that CNN-based histological tumor classification using SRH of fresh and defrosted samples of the most common tumor entities is feasible with high internal consistency.

CNN analysis achieved comparable accuracy of 90–96% in frozen samples, similar to previously published AI-based accuracy of 94.6% [14] and human-based review of SRH with an accuracy of 85% [3]. As the published CNN was mainly trained on high-quality images, the performance might have been affected by the small sample size and the lower number of high-quality diagnostic patches. A threshold of approximately 140 diagnostic patches has been identified to obtain valid CNN results [12]. An increase in the number of high-quality patches by using a larger field-of view for image acquisition in samples with impaired tissue quality might improve the predictive performance of the established CNNs. Nevertheless, the published CNN has not yet been evaluated in unfiltered, mixed-quality data or previously frozen samples.

A classification change in CNN prediction for fresh and frozen samples of the same specimen was found in one glioma and one meningioma case. One possible reason might be that SRH images at slightly different fields of view of the same sample can show different histologic features and might therefore lead to different prediction results. SRH is based on small tissue samples, similar to conventional rapid frozen sections, representing histological features from a local tumor area. Intraoperative diagnosis discrepancies between rapid frozen sections and final histological classification are well-known, especially in glioma surgery [36,37]. Therefore, it should be considered that known confounders in conventional rapid frozen sections, such as biopsy location or sample size, might also contribute to classification changes and lower overall predictive accuracy from intraoperative SRH analysis compared to final histopathological results.

Grading diffuse adult gliomas is challenging during intraoperative rapid frozen section diagnostics, as unclear cases are usually categorized as “infiltrating glioma” without further grading [3,36]. A large clinical validation study comparing SRH with standard H&E stained tissue found a lower diagnostic accuracy of 77.6% for gliomas compared to an overall accuracy of 85% [3]. Especially the occurrence of classification changes in glial tumors between intraoperative rapid frozen sections and final histopathological results are a known challenge of intraoperative tissue analysis in glioma surgery [36]. Therefore, the determination of molecular features as IDH mutation and LOH status as stable parameters in adult-type gliomas have become mandatory for molecular integrated diagnoses according to the WHO classification of brain tumors 2021 [38]. Conventional rapid frozen section analysis lacks the ability to perform molecular subtyping and molecular pathology integrated diagnosis of gliomas. AI-based SRH analysis might offer a potential solution for time-efficient intraoperative molecular subtype analysis and consequent improvement in glioma rapid diagnostics for intraoperative decision-making.

In our study, the results of the genetic tissue analysis indicate that SRH-based molecular glioma subtype classification seems to be feasible in defrosted tumor samples. Nevertheless, we observed a lower accuracy of 70–80% for molecular classification compared to the histological entity prediction Therefore, the results should be interpreted with caution. Observed changes in the molecular subtype prediction might have been associated with small samples and field-of views lacking certain diagnostical features necessary for complex AI analysis limiting a profound prediction, especially in borderline cases with lower CNN prediction levels.

Although further training of established AI tools would benefit most from SRH images of rare tumor entities, our proof-of concept study included the most common neurooncological tumor classes represented in the CNN. For rare tumor classes like pilocytic astrocytoma, ependymoma, and medulloblastoma, a lower predictive class accuracy of 50–75% for CNN analysis was reported [14]. Therefore, the use of these rare tumor entities would have potentially impaired the validity of our results. Nonetheless, the analysis of defrosted samples from rare tumor classes needs be further tested to validate our promising findings.

Beyond the limited sample number, the generalizability of our results may be affected by the varied freezing techniques used in neurooncological research. Even though we used a simple standard freezing process in the prospective patient cohort, a high image quality without significant freezing artifacts was achieved and confirmed in the biobank dataset with samples that underwent a different freezing method. These results indicate that SRH might be feasible even for tissues that have undergone different freezing protocols, making data pooling from different centers and biobanks feasible.

In the biobank dataset, different tissue samples of the same specimen were scanned using the SRH microscope, both freshly on the day of surgery and after defrosting. The freshly scanned tissue sample from the squash preparation was not available for further analysis. Using an additional tissue sample of the same specimen may capture different histological features and areas of a tumor. Nevertheless, the CNN prediction showed high internal consistency between scans of the two different samples.

One limitation of our study is the relatively low number of specimens included in the patient dataset, even though further analysis of the biobank dataset confirmed the reliability results. Our findings suggest that using frozen tissue samples to train larger CNNs seems to be generally feasible, warranting further evaluation in follow-up studies focusing on rare tumor entities.

While the CNN for tumor entity prediction in fresh and previously frozen tissue achieved high accuracy of up to 96%, the results of the molecular glioma classification showed a lower accuracy of 70–80%. Therefore, these results should be interpreted with caution. To assess applicability of molecular classification using SRH in previously frozen tissue samples, the performance of the CNN requires further validation, preferably in a larger cohort of different glial tumors with larger samples and fields of view.

Furthermore, as the CNN used for tumor entity prediction is trained to capture typical histomorphological features, the performance of this classifier might be more likely to be impaired by freezing artifacts while using previously frozen tumor tissue. In comparison, a CNN-based on biomolecular components for molecular tumor classification might be less prone to freezing artifacts but might be affected by changes in the biochemical composition in the raw spectroscopic data due to e.g., long-term freezing.

Another limitation of our study is that image quality scores were assessed objectively by an experienced neuropathologist with potential observer related bias. Objective quantitative image quality assessment, as introduced in digital pathology for whole slide image analysis [39,40] would have been beneficial. Automated image quality assessment was not feasible for our study as it is has not yet been routinely established for SRH imaging. Visual image analysis is time-consuming, expensive, and constrained suitable for large datasets that are needed for further CNN training. Therefore, evaluation and establishment of automated image quality assessment methods as used in digital pathology could avoid observer related bias and enable objective time and cost-efficient image quality control in the future.

In the first portable clinically certified SRH microscope, two Raman shifts are implemented, which are intrinsic to the medical device for in vitro diagnostic use and cannot be adapted based on the IVD MDR. Therefore, further analysis of a broader Raman spectrum is not possible based on the Raman microscope in clinical use and the presented results are mostly valid for the implemented two Raman shifts, representing the most important biomolecular peaks to enable virtual HE-like diagnostics while analyzing the most common tissue types.

Our results suggest that SRH is generally feasible for defrosted tumor tissue. Therefore, in clinical practice, freshly scanned squashed preparations or additional tumor samples can be frozen and stored for possible further SRH imaging. Implementation of the freezing workflow offers the opportunity to acquire at least double the amount of data when defrosting the samples to augment datasets for further development of diagnostic AI-based tools in a reasonable timeframe. Nevertheless, potential pitfalls such as varying freezing and defrosting processes and the stability of freezing conditions must be considered. Furthermore, regular random SRH image quality checks for defrosted tissue samples should be implemented using subjective or objective image assessment.

## 5. Conclusions

The general image quality of SRH images of different neurooncological tumor classes was found to be highly concordant between freshly acquired and previously frozen tumor samples. Potential freezing artifacts that might preclude histological and molecular pathological diagnosis were rare. CNN-based analyses of SRH images demonstrated high test–retest reliability despite samples being frozen and defrosted in between. These findings suggest that SRH databases could be augmented with rare entities derived from frozen tumor biobank samples to widen the diagnostic spectrum and further improve the accuracy of CNN prediction tools in the future.

## Data Availability

The datasets used during the current study are available from the corresponding author on reasonable request.

## Supplementary Materials

The following supporting information can be downloaded at: https://www.mdpi.com/article/10.3390/diagnostics14232701/s1, Table S1: Patients characteristics and histopathological results for the specimens in the patient and biobank dataset. mut = mutated, wt = wildtype.

## References

[1] Orringer D.A., Pandian B., Niknafs Y.S., Hollon T.C., Boyle J., Lewis S., Garrard M., Hervey-Jumper S.L., Garton H.J.L., Maher C.O. (2017). Rapid Intraoperative Histology of Unprocessed Surgical Specimens via Fibre-Laser-Based Stimulated Raman Scattering Microscopy. Nat. Biomed. Eng..

[2] Freudiger C.W., Min W., Saar B.G., Lu S., Holtom G.R., He C., Tsai J.C., Kang J.X., Xie X.S. (2008). Label-Free Biomedical Imaging with High Sensitivity by Stimulated Raman Scattering Microscopy. Science.

[3] Movahed-Ezazi M., Nasir-Moin M., Fang C., Pizzillo I., Galbraith K., Drexler S., Krasnozhen-Ratush O.A., Shroff S., Zagzag D., William C. (2023). Clinical Validation of Stimulated Raman Histology for Rapid Intraoperative Diagnosis of Central Nervous System Tumors. Mod. Pathol..

[4] Eichberg D.G., Shah A.H., Di L., Semonche A.M., Jimsheleishvili G., Luther E.M., Sarkiss C.A., Levi A.D., Gultekin S.H., Komotar R.J. (2019). Stimulated Raman Histology for Rapid and Accurate Intraoperative Diagnosis of CNS Tumors: Prospective Blinded Study. J. Neurosurg..

[5] Einstein E.H., Ablyazova F., Rosenberg A., Harshan M., Wahl S., Har-El G., Constantino P.D., Ellis J.A., Boockvar J.A., Langer D.J. (2022). Stimulated Raman Histology Facilitates Accurate Diagnosis in Neurosurgical Patients: A One-to-One Noninferiority Study. J. Neurooncol..

[6] Straehle J., Erny D., Neidert N., Henrik Heiland D., El Rahal A., Sacalean V., Steybe D., Schmelzeisen R., Vlachos A., Mizaikoff B. (2022). Neuropathological Interpretation of Stimulated Raman Histology Images of Brain and Spine Tumors: Part B. Neurosurg. Rev..

[7] Wadiura L.I., Kiesel B., Roetzer-Pejrimovsky T., Mischkulnig M., Vogel C.C., Hainfellner J.A., Matula C., Freudiger C.W., Orringer D.A., Wöhrer A. (2022). Toward Digital Histopathological Assessment in Surgery for Central Nervous System Tumors Using Stimulated Raman Histology. Neurosurg. Focus.

[8] Hollon T.C., Lewis S., Pandian B., Niknafs Y.S., Garrard M.R., Garton H., Maher C.O., McFadden K., Snuderl M., Lieberman A.P. (2018). Rapid Intraoperative Diagnosis of Pediatric Brain Tumors Using Stimulated Raman Histology. Cancer Res..

[9] Di L., Eichberg D.G., Park Y.J., Shah A.H., Jamshidi A.M., Luther E.M., Lu V.M., Komotar R.J., Ivan M.E., Gultekin S.H. (2021). Rapid Intraoperative Diagnosis of Meningiomas Using Stimulated Raman Histology. World Neurosurg..

[10] Pekmezci M., Morshed R.A., Chunduru P., Pandian B., Young J., Villanueva-Meyer J.E., Tihan T., Sloan E.A., Aghi M.K., Molinaro A.M. (2021). Detection of Glioma Infiltration at the Tumor Margin Using Quantitative Stimulated Raman Scattering Histology. Sci. Rep..

[11] Jiang C., Bhattacharya A., Linzey J.R., Joshi R.S., Cha S.J., Srinivasan S., Alber D., Kondepudi A., Urias E., Pandian B. (2022). Rapid Automated Analysis of Skull Base Tumor Specimens Using Intraoperative Optical Imaging and Artificial Intelligence. Neurosurgery.

[12] Reinecke D., Ruess D., Meissner A.-K., Fürtjes G., von Spreckelsen N., Ion-Margineau A., Khalid F., Blau T., Stehle T., Al-Shughri A. (2024). Streamlined Intraoperative Brain Tumor Classification and Molecular Subtyping in Stereotactic Biopsies Using Stimulated Raman Histology and Deep Learning. Clin. Cancer Res..

[13] Reinecke D., Spreckelsen N., Mawrin C., Ion-Margineanu A., Fürtjes G., Jünger S.T., Khalid F., Freudiger C.W., Timmer M., Ruge M.I. (2022). Novel Rapid Intraoperative Qualitative Tumor Detection by a Residual Convolutional Neural Network Using Label-Free Stimulated Raman Scattering Microscopy. Acta Neuropathol. Commun..

[14] Hollon T.C., Pandian B., Adapa A.R., Urias E., Save A.V., Khalsa S.S.S., Eichberg D.G., D’Amico R.S., Farooq Z.U., Lewis S. (2020). Near Real-Time Intraoperative Brain Tumor Diagnosis Using Stimulated Raman Histology and Deep Neural Networks. Nat. Med..

[15] Hollon T., Jiang C., Chowdury A., Nasir-Moin M., Kondepudi A., Aabedi A., Adapa A., Al-Holou W., Heth J., Sagher O. (2023). Artificial-Intelligence-Based Molecular Classification of Diffuse Gliomas Using Rapid, Label-Free Optical Imaging. Nat. Med..

[16] Hollon T.C., Pandian B., Urias E., Save A.V., Adapa A.R., Srinivasan S., Jairath N.K., Farooq Z., Marie T., Al-Holou W.N. (2021). Rapid, Label-Free Detection of Diffuse Glioma Recurrence Using Intraoperative Stimulated Raman Histology and Deep Neural Networks. Neuro Oncol..

[17] Fürtjes G., Reinecke D., Von Spreckelsen N., Meißner A.-K., Rueß D., Timmer M., Freudiger C., Ion-Margineanu A., Khalid F., Watrinet K. (2023). Intraoperative Microscopic Autofluorescence Detection and Characterization in Brain Tumors Using Stimulated Raman Histology and Two-Photon Fluorescence. Front. Oncol..

[18] Neidert N., Straehle J., Erny D., Sacalean V., El Rahal A., Steybe D., Schmelzeisen R., Vlachos A., Reinacher P.C., Coenen V.A. (2022). Stimulated Raman Histology in the Neurosurgical Workflow of a Major European Neurosurgical Center—Part A. Neurosurg. Rev..

[19] Hollon T.C., Orringer D.A. (2020). An Automated Tissue-to-Diagnosis Pipeline Using Intraoperative Stimulated Raman Histology and Deep Learning. Mol. Cell Oncol..

[20] Hollon T., Orringer D.A. (2021). Label-Free Brain Tumor Imaging Using Raman-Based Methods. J. Neurooncol..

[21] Shrestha P., Kneepkens R., van Elswijk G., Vrijnsen J., Ion R., Verhagen D., Abels E., Vossen D., Hulsken B. (2015). Objective and Subjective Assessment of Digital Pathology Image Quality. AIMS Med. Sci..

[22] Hashimoto N., Bautista P.A., Yamaguchi M., Ohyama N., Yagi Y. (2012). Referenceless Image Quality Evaluation for Whole Slide Imaging. J. Pathol. Inform..

[23] Forero C.G. (2014). Cronbach’s Alpha. Encyclopedia of Quality of Life and Well-Being Research.

[24] Khonglah Y., Lyngdoh B.S., Kakati A., Mishra J., Al Aman M.M., Phukan P. (2021). Intraoperative Diagnosis of Central Nervous System Tumors: Challenges, Errors, Lessons Learned, and the Surgeon’s Perspective. Cureus.

[25] Meißner A.-K., Goldbrunner R., Neuschmelting V. (2024). Intraoperative Stimulated Raman Histology for Personalized Brain Tumor Surgery. Chirurgie.

[26] Klamminger G.G., Gérardy J.-J., Jelke F., Mirizzi G., Slimani R., Klein K., Husch A., Hertel F., Mittelbronn M., Kleine-Borgmann F.B. (2021). Application of Raman Spectroscopy for Detection of Histologically Distinct Areas in Formalin-Fixed Paraffin-Embedded Glioblastoma. Neurooncol. Adv..

[27] Kalkanis S.N., Kast R.E., Rosenblum M.L., Mikkelsen T., Yurgelevic S.M., Nelson K.M., Raghunathan A., Poisson L.M., Auner G.W. (2014). Raman Spectroscopy to Distinguish Grey Matter, Necrosis, and Glioblastoma Multiforme in Frozen Tissue Sections. J. Neurooncol..

[28] Kast R.E., Auner G.W., Rosenblum M.L., Mikkelsen T., Yurgelevic S.M., Raghunathan A., Poisson L.M., Kalkanis S.N. (2014). Raman Molecular Imaging of Brain Frozen Tissue Sections. J. Neurooncol..

[29] Draux F., Gobinet C., Sulé-Suso J., Trussardi A., Manfait M., Jeannesson P., Sockalingum G.D. (2010). Raman Spectral Imaging of Single Cancer Cells: Probing the Impact of Sample Fixation Methods. Anal. Bioanal. Chem..

[30] Huang Z., McWilliams A., Lam S., English J., McLean D., Lui H., Zeng H. (2003). Effect of Formalin Fixation on the Near-Infrared Raman Spectroscopy of Normal and Cancerous Human Bronchial Tissues. Int. J. Oncol..

[31] Desciak E.B., Maloney M.E. (2000). Artifacts in Frozen Section Preparation. Dermatol. Surg..

[32] Nackenhorst M.C., Kasiri M., Gollackner B., Regele H. (2022). Ex Vivo Fluorescence Confocal Microscopy: Chances and Changes in the Analysis of Breast Tissue. Diagn. Pathol..

[33] Shabihkhani M., Lucey G.M., Wei B., Mareninov S., Lou J.J., Vinters H.V., Singer E.J., Cloughesy T.F., Yong W.H. (2014). The Procurement, Storage, and Quality Assurance of Frozen Blood and Tissue Biospecimens in Pathology, Biorepository, and Biobank Settings. Clin. Biochem..

[34] Ericsson C., Franzén B., Nistér M. (2006). Frozen Tissue Biobanks. Tissue Handling, Cryopreservation, Extraction, and Use for Proteomic Analysis. Acta Oncol..

[35] Estrada L.I., Robinson A.A., Amaral A.C., Giannaris E.L., Heyworth N.C., Mortazavi F., Ngwenya L.B., Roberts D.E., Cabral H.J., Killiany R.J. (2017). Evaluation of Long-Term Cryostorage of Brain Tissue Sections for Quantitative Histochemistry. J. Histochem. Cytochem..

[36] Harms J.W.A., Streckert E.M.S., Kiolbassa N.M., Thomas C., Grauer O., Oertel M., Eich H.T., Stummer W., Paulus W., Brokinkel B. (2023). Confounders of Intraoperative Frozen Section Pathology during Glioma Surgery. Neurosurg. Rev..

[37] Yadav M., Sharma P., Singh V., Tewari R., Mishra P.S., Roy K. (2022). An Audit of Diagnostic Disparity between Intraoperative Frozen Section Diagnosis and Final Histopathological Diagnosis of Central Nervous System Lesions at a Tertiary Care Center. J. Lab. Physicians.

[38] Louis D.N., Perry A., Wesseling P., Brat D.J., Cree I.A., Figarella-Branger D., Hawkins C., Ng H.K., Pfister S.M., Reifenberger G. (2021). The 2021 WHO Classification of Tumors of the Central Nervous System: A Summary. Neuro Oncol..

[39] Wright A.I., Dunn C.M., Hale M., Hutchins G.G.A., Treanor D.E. (2021). The Effect of Quality Control on Accuracy of Digital Pathology Image Analysis. IEEE J. Biomed. Health Inform..

[40] Hosseini M.S., Brawley-Hayes J.A.Z., Zhang Y., Chan L., Plataniotis K., Damaskinos S. (2020). Focus Quality Assessment of High-Throughput Whole Slide Imaging in Digital Pathology. IEEE Trans. Med. Imaging.

