# Supplementary material for: Image Quality Assessment and Reliability Analysis of Artificial Intelligence-Based Tumor Classification of Stimulated Raman Histology of Tumor Biobank Samples
Source: Diagnostics (Basel). 2024 Nov 30;14(23):2701. doi: 10.3390/diagnostics14232701 (PMC11640452; doi:10.3390/diagnostics14232701)
Supplement: Supplementary file 1 [file diagnostics-14-02701-s001.zip › diagnostics-3306856-supplementary.pdf]

Supplementary Materials

Table S1. Patients characteristics and histopathological results for the specimens in the patient and biobank dataset. *mut = mutated, wt = wildtype.*

Patients Characteristics	Patient Dataset (n = 25)	Biobank Dataset (n = 30)
Gender (female/male)	11/14	21/9
Age at surgery (years) (mean ± SD)	56 ± 14	58 ± 15

Histopathological result	Patient dataset		Biobank dataset	
	Patients	Images	Patients	Images
Meningioma	5	10	5	10
• CNS WHO 1	4	8	5	10
• CNS WHO 2	1	2	0	0
Diffuse adult glioma	9	18	13	26
• astrocytoma, IDH mut, CNS WHO 2	1	2	1	2
• astrocytoma, IDH mut, CNS WHO 3	0	0	2	4
• astrocytoma, IDH mut, CNS WHO 4	1	2	0	0
• glioma, IDH wt, CNS WHO 4	7	14	10	20
Pituitary adenoma	4	8	5	10
Metastases	4	8	4	8
• carcinoma	3	6	4	8
• melanoma	1	2	0	0
Schwannoma	3	6	3	6
